# 5-Fluoro-*N*-(2-methyl-3-oxo-1-thia-4-aza­spiro­[4.5]dec-4-yl)-3-phenyl-1*H*-indole-2-carboxamide

**DOI:** 10.1107/S1600536813018576

**Published:** 2013-07-10

**Authors:** Sevim Türktekin Çelikesir, Mehmet Akkurt, Gökçe Cihan Üstündağ, Orhan Büyükgüngör

**Affiliations:** a Department of Physics, Faculty of Sciences, Erciyes University, 38039 Kayseri, Turkey; b Department of Pharmaceutical Chemistry, Faculty of Pharmacy, Istanbul University, 34116 Beyazit, Istanbul, Turkey; c Department of Physics, Faculty of Arts and Sciences, Ondokuz Mayıs University, 55139 Samsun, Turkey

## Abstract

In the title compound, C_24_H_24_FN_3_O_2_S, the 1,3-thia­zolidine ring adopts an envelope conformation with the S atom as the flap, while the cyclo­hexane ring is in a chair conformation. In the crystal, mol­ecules are linked by N—H⋯O and C—H⋯F hydrogen bonds, forming a three-dimensional network. The unit cell contains six voids of 57 Å^3^, but the residual electron density (highest peak = 0.23 e Å^−3^ and deepest hole = −0.19 e Å^−3^) in the difference Fourier map suggests no solvent mol­ecule occupies this void.

## Related literature

For the anti­tubercular and anti­viral activity of variously substituted *N*-(1-thia-4-aza­spiro­[4.5]dec-4-yl)carboxamides, see: Cihan-Üstündağ & Çapan (2012 ▶); Göktas *et al.* (2012 ▶); Güzel *et al.* (2006 ▶); Ulusoy (2002 ▶); Vanderlinden *et al.* (2010 ▶). For puckering analysis, see: Cremer & Pople (1975 ▶).
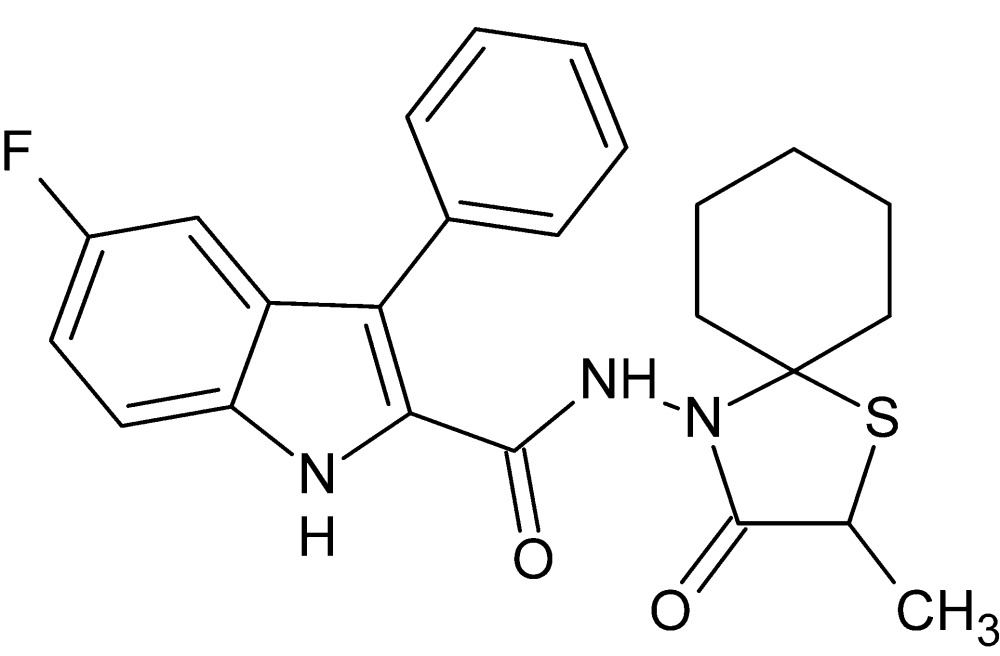


## Experimental

### 

#### Crystal data

C_24_H_24_FN_3_O_2_S
*M* *_r_* = 437.53
Hexagonal, 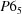

*a* = 13.2082 (18) Å
*c* = 23.584 (4) Å
*V* = 3563.2 (13) Å^3^
*Z* = 6
Mo *K*α radiation
μ = 0.17 mm^−1^
*T* = 296 K
0.68 × 0.49 × 0.40 mm

#### Data collection

Stoe IPDS 2 diffractometer
Absorption correction: integration (*X-RED32*; Stoe & Cie, 2002 ▶) *T*_min_ = 0.905, *T*_max_ = 0.935
37961 measured reflections
4922 independent reflections
3348 reflections with *I* > 2σ(*I*)
*R*_int_ = 0.059

#### Refinement

*R*[*F* ^2^ > 2σ(*F* ^2^)] = 0.042
*wR*(*F* ^2^) = 0.100
*S* = 0.96
4922 reflections
288 parameters
4 restraints
H atoms treated by a mixture of independent and constrained refinement
Δρ_max_ = 0.23 e Å^−3^
Δρ_min_ = −0.19 e Å^−3^
Absolute structure: Flack (1983 ▶), 2399 Freidel pairs
Flack parameter: −0.01 (8)

### 

Data collection: *X-AREA* (Stoe & Cie, 2002 ▶); cell refinement: *X-AREA*; data reduction: *X-RED32* (Stoe & Cie, 2002 ▶); program(s) used to solve structure: *SHELXS97* (Sheldrick, 2008 ▶); program(s) used to refine structure: *SHELXL97* (Sheldrick, 2008 ▶); molecular graphics: *ORTEP-3 for Windows* (Farrugia, 2012 ▶); software used to prepare material for publication: *WinGX* (Farrugia, 2012 ▶).

## Supplementary Material

Crystal structure: contains datablock(s) global, I. DOI: 10.1107/S1600536813018576/qm2099sup1.cif

Structure factors: contains datablock(s) I. DOI: 10.1107/S1600536813018576/qm2099Isup2.hkl

Supplementary material file. DOI: 10.1107/S1600536813018576/qm2099Isup3.cml

Additional supplementary materials:  crystallographic information; 3D view; checkCIF report

## Figures and Tables

**Table 1:** Hydrogen-bond geometry (Å, °)

*D*—H⋯*A*	*D*—H	H⋯*A*	*D*⋯*A*	*D*—H⋯*A*
N1—H1*A*⋯O2^i^	0.86 (3)	2.08 (3)	2.903 (4)	160 (2)
N2—H2*A*⋯O1^ii^	0.85 (2)	2.07 (2)	2.760 (3)	137 (2)
C10—H10*A*⋯F1^iii^	0.93	2.54	3.453 (5)	167

Symmetry codes: (i) 
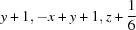
; (ii) 
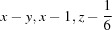
; (iii) 
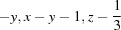
.

**Fig. 1.**
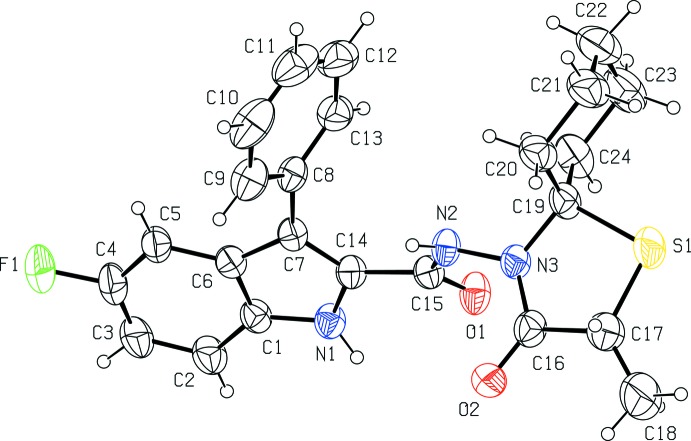
The structure of the title molecule with the atom labelling scheme. Displacement ellipsoids for non-H atoms are drawn at the 30% probability level.

**Fig. 2.**
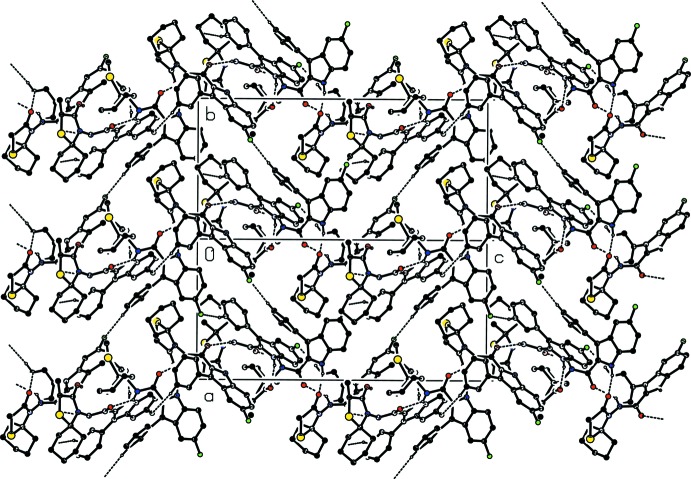
View of the packing and hydrogen bondings of the title compound, down the [110]-axis. H atoms not participating in hydrogen bonding have been omitted for clarity.

## supplementary crystallographic information

### Comment

Despite remarkable advances, tuberculosis and viral diseases continue to be the leading causes of death worldwide. Recent research on variously substituted *N*-(1-thia-4-azaspiro[4.5]dec-4-yl)carboxamides has revealed encouraging antitubercular (Cihan-Üstündağ & Çapan, 2012; Güzel *et al.* 2006; Ulusoy, 2002) and antiviral (Göktas *et al.*, 2012; Vanderlinden *et al.*, 2010) activity. Full characterization of the active core may yield invaluable data for the design of relevant compounds with enhanced action. Thus, we herein report the X-ray diffraction analysis of the title compound (I).

In (I), (Fig. 1), the 1,3 thiazolidine ring (S1/N3/C16/C17/C19) adopts an envelope conformation [the puckering parameters (Cremer & Pople, 1975) are Q(2) = 0.228 (3) Å and φ(2) = 351.3 (8)°] with the S1 atom as the flap atom. The cyclohexane ring (C19–C24) exhibits a chair conformation with the puckering parameters of Q_T_ = 0.554 (4) Å, θ = 180.0° and φ = 180 (15)°. The indole ring system (N1/C1–C7/C14) is essentially planar, with the maximum deviations of 0.014 (2) Å for N1 and 0.012 (3) Å for C5. The phenyl (C8–C13) and 1,3-thiazolidine (S1/N3/C16/C17/C19) rings are inclined at the dihedral angles of 56.14 (15) and 57.03 (12) °, respectively, to the indole ring system. The torsion angle of the N3–N2–C15–C14 bridge between the indole ring and the thiazolidine ring system is -165.8 (2) °.

In the crystal structure, N—H···O and C—H···F hydrogen bonds connect the adjacent molecules to each other, forming a three dimensional network. In addtion, π-π and C—H···π interactions are not observed.

### Experimental

A mixture of 5-fluoro-3-phenyl-1*H*-indole-2-carbohydrazide (0.0025 mol), cyclohexanone (0.003 mol) and 2-mercaptopropionic acid (0.01 mol) was refluxed in 20 ml dry benzene for 5 h using a Dean-Stark water separator. Excess benzene was evaporated *in vacuo*. The resulting residue was triturated with saturated NaHCO_3_ solution until CO_2_ evolution ceased and was refrigerated overnight. The solid thus obtained was washed with water, filtered, dried, and recrystallized from ethanol.

Yield: 77%, mp.: 507–509 K. IR(KBr): υ_max_ 3227 (N—H), 1690 (C=O), 1662 (C=O) cm^-1. 1^H-NMR (DMSO-d_6_/500 MHz): δ 1.03–1.09 (m, 1H, CH_2_-*sp*.*), 1.32–1.45 (m, 5H, 2-CH_3_, CH_2_-*sp*.), 1.55–1.77 (m, 7H, CH_2_-*sp*.), 3.87 (br. d, 1H, J=6.3 Hz, C2—H -*sp*.), 7.15 (td, 1H, J=9.4, 2.4 Hz, H6-ind.), 7.23 (dd, 1H, J=9.7, 1.9 Hz, H4-ind.), 7.35 (t, 1H, J=7.3 Hz, 3-C_6_H_5_(H4)-ind.), 7.45 (t, 2H, J=7.3 Hz, 3-C_6_H_5_(H3,H5)-ind.), 7.50–7.57 (m, 3H, H7, 3-C_6_H_5_ (H2,H6)-ind.), 10.06 (s, 1H, CONH), 12.00 (s, 1H, NH) p.p.m.. MS (APCI+) m/*z*(%) 438 ((*M*+H)^+^, 43), (APCI–) m/*z*(%) 436 ((M—H)^-^, 100). Analysis calculated for C_24_H_24_FN_3_O_2_S: C 65.88, H 5.53, N 9.60, S 7.33%. Found: C 65.55, H 5.20, N 9.44, S 7.69%. (**sp*.=spirodecane, br.=broad, ind.=indole).

### Refinement

H atoms bonded to C atoms were positioned geometrically with C—H = 0.93 - 0.98 Å, and refined using a riding model with *U*_iso_(H) = 1.2 or 1.5*U*_eq_(C). The H atoms of the two amide groups were found in a difference Fourier map, restrained with N—H = 0.86 (2) Å and refined with *U*_iso_ = 1.2*U*_eq_(N). The unit cell contains six voids of 57 Å^3^, but the residual electron density (highest peak = 0.23 e.Å^-3^ and deepest hole = -0.19 e.Å^-3^) in the difference Fourier map suggests no solvent molecule occupies this void.

### Crystal data

**Table:**

C_24_H_24_FN_3_O_2_S	*D*_x_ = 1.223 Mg m^−^^3^
*M**_r_* = 437.53	Mo *K*α radiation, λ = 0.71073 Å
Hexagonal, *P*6_5_	Cell parameters from 3314 reflections
Hall symbol: P 65	θ = 1.7–24.3°
*a* = 13.2082 (18) Å	µ = 0.17 mm^−^^1^
*c* = 23.584 (4) Å	*T* = 296 K
*V* = 3563.2 (13) Å^3^	Prism, colourless
*Z* = 6	0.68 × 0.49 × 0.40 mm
*F*(000) = 1380	

### Data collection

**Table:**

Stoe IPDS 2 diffractometer	4922 independent reflections
Radiation source: sealed X-ray tube, 12 x 0.4 mm long-fine focus	3348 reflections with *I* > 2σ(*I*)
Plane graphite monochromator	*R*_int_ = 0.059
Detector resolution: 6.67 pixels mm^-1^	θ_max_ = 26.5°, θ_min_ = 1.8°
ω–scans	*h* = −15→16
Absorption correction: integration (*X-RED32*; Stoe & Cie, 2002)	*k* = −16→16
*T*_min_ = 0.905, *T*_max_ = 0.935	*l* = −29→29
37961 measured reflections	

### Refinement

**Table:**

Refinement on *F*^2^	Secondary atom site location: difference Fourier map
Least-squares matrix: full	Hydrogen site location: inferred from neighbouring sites
*R*[*F*^2^ > 2σ(*F*^2^)] = 0.042	H atoms treated by a mixture of independent and constrained refinement
*wR*(*F*^2^) = 0.100	*w* = 1/[σ^2^(*F*_o_^2^) + (0.0551*P*)^2^] where *P* = (*F*_o_^2^ + 2*F*_c_^2^)/3
*S* = 0.96	(Δ/σ)_max_ < 0.001
4922 reflections	Δρ_max_ = 0.23 e Å^−^^3^
288 parameters	Δρ_min_ = −0.19 e Å^−^^3^
4 restraints	Absolute structure: Flack (1983), 2399 Freidel pairs
Primary atom site location: structure-invariant direct methods	Flack parameter: −0.01 (8)

### Special details

**Table:**

Geometry. Bond distances, angles *etc*. have been calculated using the rounded fractional coordinates. All su's are estimated from the variances of the (full) variance-covariance matrix. The cell e.s.d.'s are taken into account in the estimation of distances, angles and torsion angles
Refinement. Refinement on *F*^2^ for ALL reflections except those flagged by the user for potential systematic errors. Weighted *R*-factors *wR* and all goodnesses of fit *S* are based on *F*^2^, conventional *R*-factors *R* are based on *F*, with *F* set to zero for negative *F*^2^. The observed criterion of *F*^2^ > σ(*F*^2^) is used only for calculating -*R*-factor-obs *etc*. and is not relevant to the choice of reflections for refinement. *R*-factors based on *F*^2^ are statistically about twice as large as those based on *F*, and *R*-factors based on ALL data will be even larger.

### Fractional atomic coordinates and isotropic or equivalent isotropic displacement parameters (Å^2^)

**Table:**

	*x*	*y*	*z*	*U*_iso_*/*U*_eq_	
S1	1.14962 (8)	0.40748 (8)	0.02744 (4)	0.0985 (3)	
F1	0.46476 (17)	−0.29139 (16)	0.34676 (8)	0.1106 (8)	
O1	1.03682 (16)	0.23902 (16)	0.20319 (6)	0.0747 (6)	
O2	1.05778 (17)	0.10407 (18)	0.08858 (8)	0.0808 (7)	
N1	0.8840 (2)	0.04416 (18)	0.26571 (8)	0.0635 (7)	
N2	0.91194 (19)	0.17601 (19)	0.12881 (7)	0.0608 (7)	
N3	1.00273 (18)	0.24151 (18)	0.09113 (8)	0.0609 (7)	
C1	0.7870 (2)	−0.0441 (2)	0.29191 (9)	0.0617 (9)	
C2	0.7796 (3)	−0.1096 (3)	0.33995 (11)	0.0793 (10)	
C3	0.6684 (3)	−0.1925 (3)	0.35702 (13)	0.0895 (13)	
C4	0.5723 (3)	−0.2078 (2)	0.32737 (11)	0.0792 (10)	
C5	0.5760 (2)	−0.1454 (2)	0.28088 (11)	0.0690 (9)	
C6	0.6880 (2)	−0.0611 (2)	0.26178 (9)	0.0585 (8)	
C7	0.7284 (2)	0.0200 (2)	0.21584 (9)	0.0565 (8)	
C8	0.6543 (2)	0.0362 (2)	0.17393 (9)	0.0613 (8)	
C9	0.5750 (3)	−0.0575 (3)	0.14063 (11)	0.0862 (11)	
C10	0.5091 (3)	−0.0395 (5)	0.09960 (14)	0.1120 (16)	
C11	0.5207 (4)	0.0666 (5)	0.09194 (16)	0.114 (2)	
C12	0.5970 (3)	0.1599 (4)	0.12420 (16)	0.1037 (16)	
C13	0.6620 (3)	0.1432 (3)	0.16482 (12)	0.0796 (11)	
C14	0.8473 (2)	0.0809 (2)	0.21937 (9)	0.0575 (8)	
C15	0.9402 (2)	0.1718 (2)	0.18413 (9)	0.0579 (8)	
C16	1.0671 (2)	0.1961 (3)	0.07269 (10)	0.0715 (10)	
C17	1.1532 (4)	0.2742 (3)	0.02812 (15)	0.1084 (16)	
C18	1.2528 (5)	0.2705 (5)	0.0215 (3)	0.218 (4)	
C19	1.0107 (3)	0.3462 (2)	0.06577 (10)	0.0685 (9)	
C20	0.9079 (3)	0.3128 (3)	0.02508 (12)	0.0823 (10)	
C21	0.9134 (4)	0.4214 (3)	−0.00119 (14)	0.1053 (16)	
C22	0.9213 (5)	0.5057 (4)	0.04366 (19)	0.1259 (19)	
C23	1.0202 (4)	0.5387 (3)	0.08365 (16)	0.1170 (18)	
C24	1.0156 (3)	0.4300 (3)	0.11059 (12)	0.0937 (13)	
H1A	0.9546 (16)	0.058 (2)	0.2688 (11)	0.067 (8)*	
H2	0.84590	−0.09790	0.35940	0.0950*	
H2A	0.8520 (18)	0.1166 (18)	0.1151 (9)	0.063 (8)*	
H3	0.65870	−0.23830	0.38880	0.1070*	
H5	0.50840	−0.15760	0.26270	0.0830*	
H9	0.56600	−0.13130	0.14570	0.1030*	
H10A	0.45660	−0.10190	0.07730	0.1340*	
H11A	0.47620	0.07680	0.06440	0.1370*	
H12	0.60480	0.23320	0.11880	0.1240*	
H13	0.71320	0.20640	0.18700	0.0950*	
H17	1.11130	0.23700	−0.00690	0.1300*	
H18A	1.30540	0.31180	0.05210	0.2610*	
H18B	1.28880	0.30640	−0.01390	0.2610*	
H18C	1.23510	0.19060	0.02160	0.2610*	
H20A	0.90960	0.26330	−0.00480	0.0990*	
H20B	0.83490	0.26870	0.04560	0.0990*	
H21A	0.98100	0.45960	−0.02590	0.1270*	
H21B	0.84410	0.39800	−0.02400	0.1270*	
H22A	0.93070	0.57580	0.02550	0.1510*	
H22B	0.84870	0.47080	0.06490	0.1510*	
H23A	1.01730	0.58790	0.11340	0.1410*	
H23B	1.09350	0.58330	0.06350	0.1410*	
H24A	1.08430	0.45420	0.13400	0.1130*	
H24B	0.94720	0.39070	0.13470	0.1130*	

### Atomic displacement parameters (Å^2^)

**Table:**

	*U*^11^	*U*^22^	*U*^33^	*U*^12^	*U*^13^	*U*^23^
S1	0.1004 (6)	0.0842 (5)	0.1055 (5)	0.0421 (5)	0.0484 (5)	0.0345 (4)
F1	0.0989 (14)	0.0883 (12)	0.1143 (13)	0.0241 (11)	0.0351 (11)	0.0409 (10)
O1	0.0642 (11)	0.0767 (12)	0.0567 (9)	0.0154 (10)	−0.0039 (8)	0.0082 (8)
O2	0.0818 (13)	0.0784 (13)	0.0904 (12)	0.0462 (11)	0.0147 (10)	0.0192 (11)
N1	0.0608 (14)	0.0695 (14)	0.0537 (10)	0.0276 (12)	0.0009 (10)	0.0105 (9)
N2	0.0578 (13)	0.0649 (14)	0.0453 (10)	0.0199 (11)	0.0062 (9)	0.0057 (9)
N3	0.0656 (13)	0.0643 (12)	0.0511 (9)	0.0311 (11)	0.0139 (9)	0.0098 (9)
C1	0.0725 (17)	0.0609 (15)	0.0513 (12)	0.0330 (14)	0.0071 (11)	0.0068 (11)
C2	0.088 (2)	0.0773 (19)	0.0683 (15)	0.0381 (17)	0.0029 (14)	0.0177 (13)
C3	0.104 (3)	0.083 (2)	0.0765 (16)	0.0429 (19)	0.0204 (18)	0.0353 (15)
C4	0.084 (2)	0.0590 (17)	0.0740 (16)	0.0203 (15)	0.0233 (16)	0.0131 (13)
C5	0.0689 (17)	0.0574 (15)	0.0720 (14)	0.0250 (14)	0.0138 (12)	0.0078 (12)
C6	0.0667 (16)	0.0547 (14)	0.0533 (11)	0.0297 (13)	0.0059 (11)	0.0003 (10)
C7	0.0622 (16)	0.0521 (14)	0.0495 (11)	0.0244 (12)	0.0046 (10)	−0.0012 (10)
C8	0.0562 (14)	0.0718 (17)	0.0486 (11)	0.0266 (13)	0.0102 (10)	0.0048 (11)
C9	0.0690 (19)	0.096 (2)	0.0760 (17)	0.0281 (17)	−0.0107 (14)	−0.0130 (16)
C10	0.069 (2)	0.164 (4)	0.080 (2)	0.041 (3)	−0.0156 (16)	−0.026 (2)
C11	0.093 (3)	0.188 (5)	0.081 (2)	0.085 (3)	0.008 (2)	0.023 (3)
C12	0.093 (3)	0.141 (3)	0.099 (2)	0.075 (3)	0.016 (2)	0.036 (2)
C13	0.0762 (19)	0.089 (2)	0.0787 (16)	0.0451 (17)	0.0079 (14)	0.0121 (14)
C14	0.0609 (15)	0.0605 (14)	0.0473 (11)	0.0275 (13)	0.0029 (10)	0.0031 (10)
C15	0.0623 (16)	0.0579 (14)	0.0502 (12)	0.0275 (13)	0.0005 (11)	0.0027 (10)
C16	0.0710 (18)	0.0785 (19)	0.0609 (14)	0.0343 (16)	0.0118 (12)	0.0110 (13)
C17	0.124 (3)	0.112 (3)	0.112 (2)	0.076 (2)	0.062 (2)	0.048 (2)
C18	0.226 (6)	0.162 (5)	0.314 (8)	0.134 (5)	0.208 (6)	0.127 (5)
C19	0.0854 (19)	0.0647 (16)	0.0541 (12)	0.0366 (14)	0.0192 (12)	0.0118 (11)
C20	0.100 (2)	0.0845 (19)	0.0726 (15)	0.0537 (18)	0.0079 (15)	0.0168 (14)
C21	0.134 (3)	0.113 (3)	0.092 (2)	0.079 (2)	0.019 (2)	0.036 (2)
C22	0.178 (4)	0.115 (3)	0.127 (3)	0.105 (3)	0.058 (3)	0.046 (3)
C23	0.176 (4)	0.081 (2)	0.103 (3)	0.071 (3)	0.032 (3)	0.0072 (19)
C24	0.135 (3)	0.0724 (19)	0.0700 (16)	0.049 (2)	0.0241 (17)	0.0028 (13)

### Geometric parameters (Å, º)

**Table:**

S1—C17	1.785 (4)	C17—C18	1.350 (9)
S1—C19	1.831 (4)	C19—C24	1.508 (4)
F1—C4	1.370 (4)	C19—C20	1.536 (5)
O1—C15	1.219 (3)	C20—C21	1.531 (5)
O2—C16	1.218 (4)	C21—C22	1.501 (6)
N1—C1	1.374 (3)	C22—C23	1.489 (8)
N1—C14	1.378 (3)	C23—C24	1.543 (5)
N2—N3	1.392 (3)	C2—H2	0.9300
N2—C15	1.366 (3)	C3—H3	0.9300
N3—C16	1.335 (4)	C5—H5	0.9300
N3—C19	1.461 (3)	C9—H9	0.9300
N1—H1A	0.86 (3)	C10—H10A	0.9300
N2—H2A	0.85 (2)	C11—H11A	0.9300
C1—C6	1.404 (4)	C12—H12	0.9300
C1—C2	1.399 (4)	C13—H13	0.9300
C2—C3	1.382 (5)	C17—H17	0.9800
C3—C4	1.373 (6)	C18—H18A	0.9600
C4—C5	1.358 (4)	C18—H18B	0.9600
C5—C6	1.409 (4)	C18—H18C	0.9600
C6—C7	1.426 (3)	C20—H20A	0.9700
C7—C14	1.363 (4)	C20—H20B	0.9700
C7—C8	1.480 (4)	C21—H21A	0.9700
C8—C13	1.382 (4)	C21—H21B	0.9700
C8—C9	1.396 (4)	C22—H22A	0.9700
C9—C10	1.399 (6)	C22—H22B	0.9700
C10—C11	1.344 (8)	C23—H23A	0.9700
C11—C12	1.368 (7)	C23—H23B	0.9700
C12—C13	1.376 (6)	C24—H24A	0.9700
C14—C15	1.471 (3)	C24—H24B	0.9700
C16—C17	1.513 (5)		
			
C17—S1—C19	94.27 (18)	C21—C22—C23	112.9 (5)
C1—N1—C14	108.2 (2)	C22—C23—C24	111.6 (3)
N3—N2—C15	117.8 (2)	C19—C24—C23	111.2 (2)
N2—N3—C16	118.2 (2)	C1—C2—H2	122.00
N2—N3—C19	118.8 (3)	C3—C2—H2	122.00
C16—N3—C19	122.1 (2)	C2—C3—H3	120.00
C1—N1—H1A	126.5 (16)	C4—C3—H3	120.00
C14—N1—H1A	121.9 (17)	C4—C5—H5	122.00
N3—N2—H2A	116.4 (15)	C6—C5—H5	122.00
C15—N2—H2A	118.2 (15)	C8—C9—H9	120.00
C2—C1—C6	122.6 (3)	C10—C9—H9	120.00
N1—C1—C2	129.5 (3)	C9—C10—H10A	119.00
N1—C1—C6	107.9 (2)	C11—C10—H10A	120.00
C1—C2—C3	116.4 (3)	C10—C11—H11A	120.00
C2—C3—C4	120.4 (3)	C12—C11—H11A	119.00
F1—C4—C5	117.8 (3)	C11—C12—H12	121.00
C3—C4—C5	124.9 (3)	C13—C12—H12	121.00
F1—C4—C3	117.3 (2)	C8—C13—H13	119.00
C4—C5—C6	116.3 (3)	C12—C13—H13	119.00
C1—C6—C5	119.4 (2)	S1—C17—H17	102.00
C1—C6—C7	107.2 (2)	C16—C17—H17	102.00
C5—C6—C7	133.4 (3)	C18—C17—H17	102.00
C6—C7—C14	106.6 (2)	C17—C18—H18A	110.00
C8—C7—C14	127.3 (2)	C17—C18—H18B	109.00
C6—C7—C8	126.1 (2)	C17—C18—H18C	109.00
C9—C8—C13	117.3 (3)	H18A—C18—H18B	110.00
C7—C8—C9	120.3 (2)	H18A—C18—H18C	109.00
C7—C8—C13	122.4 (2)	H18B—C18—H18C	109.00
C8—C9—C10	119.6 (4)	C19—C20—H20A	109.00
C9—C10—C11	120.9 (4)	C19—C20—H20B	109.00
C10—C11—C12	120.9 (5)	C21—C20—H20A	109.00
C11—C12—C13	118.7 (4)	C21—C20—H20B	109.00
C8—C13—C12	122.6 (3)	H20A—C20—H20B	108.00
N1—C14—C7	110.2 (2)	C20—C21—H21A	109.00
N1—C14—C15	116.0 (2)	C20—C21—H21B	109.00
C7—C14—C15	133.8 (2)	C22—C21—H21A	109.00
O1—C15—N2	122.0 (2)	C22—C21—H21B	109.00
N2—C15—C14	116.1 (2)	H21A—C21—H21B	108.00
O1—C15—C14	121.9 (2)	C21—C22—H22A	109.00
O2—C16—N3	125.4 (3)	C21—C22—H22B	109.00
O2—C16—C17	124.1 (3)	C23—C22—H22A	109.00
N3—C16—C17	110.5 (3)	C23—C22—H22B	109.00
C16—C17—C18	118.0 (4)	H22A—C22—H22B	108.00
S1—C17—C16	107.3 (3)	C22—C23—H23A	109.00
S1—C17—C18	122.8 (4)	C22—C23—H23B	109.00
S1—C19—C20	111.40 (19)	C24—C23—H23A	109.00
S1—C19—C24	110.8 (2)	C24—C23—H23B	109.00
N3—C19—C20	110.4 (2)	H23A—C23—H23B	108.00
S1—C19—N3	101.7 (2)	C19—C24—H24A	109.00
N3—C19—C24	111.3 (2)	C19—C24—H24B	109.00
C20—C19—C24	110.9 (3)	C23—C24—H24A	109.00
C19—C20—C21	111.3 (3)	C23—C24—H24B	109.00
C20—C21—C22	111.3 (3)	H24A—C24—H24B	108.00
			
C19—S1—C17—C16	−16.7 (3)	C5—C6—C7—C14	178.9 (3)
C19—S1—C17—C18	−158.5 (4)	C5—C6—C7—C8	0.6 (4)
C17—S1—C19—N3	18.3 (2)	C1—C6—C7—C14	0.5 (3)
C17—S1—C19—C20	−99.3 (2)	C6—C7—C8—C9	−58.0 (4)
C17—S1—C19—C24	136.7 (3)	C6—C7—C14—N1	−1.2 (3)
C1—N1—C14—C15	−177.1 (2)	C6—C7—C14—C15	176.9 (3)
C1—N1—C14—C7	1.4 (3)	C14—C7—C8—C9	124.0 (3)
C14—N1—C1—C6	−1.0 (3)	C14—C7—C8—C13	−54.3 (4)
C14—N1—C1—C2	179.2 (3)	C8—C7—C14—N1	177.1 (2)
N3—N2—C15—O1	13.9 (4)	C8—C7—C14—C15	−4.8 (5)
C15—N2—N3—C16	76.9 (3)	C6—C7—C8—C13	123.7 (3)
C15—N2—N3—C19	−113.8 (3)	C7—C8—C13—C12	177.1 (3)
N3—N2—C15—C14	−165.8 (2)	C9—C8—C13—C12	−1.2 (5)
C16—N3—C19—C24	−135.4 (3)	C13—C8—C9—C10	1.0 (5)
N2—N3—C16—O2	−4.5 (4)	C7—C8—C9—C10	−177.4 (3)
C16—N3—C19—S1	−17.3 (3)	C8—C9—C10—C11	−0.3 (6)
N2—N3—C16—C17	174.6 (2)	C9—C10—C11—C12	−0.2 (7)
C19—N3—C16—C17	5.7 (4)	C10—C11—C12—C13	0.0 (7)
N2—N3—C19—C24	55.8 (4)	C11—C12—C13—C8	0.8 (6)
C19—N3—C16—O2	−173.4 (3)	C7—C14—C15—N2	−23.2 (4)
N2—N3—C19—S1	173.89 (16)	C7—C14—C15—O1	157.1 (3)
C16—N3—C19—C20	101.1 (3)	N1—C14—C15—O1	−24.9 (4)
N2—N3—C19—C20	−67.8 (3)	N1—C14—C15—N2	154.8 (2)
C6—C1—C2—C3	−0.6 (4)	O2—C16—C17—C18	−27.3 (6)
C2—C1—C6—C7	−179.9 (3)	N3—C16—C17—S1	9.7 (3)
N1—C1—C2—C3	179.1 (3)	N3—C16—C17—C18	153.6 (4)
N1—C1—C6—C5	−178.4 (2)	O2—C16—C17—S1	−171.2 (3)
C2—C1—C6—C5	1.4 (4)	S1—C19—C20—C21	−68.8 (3)
N1—C1—C6—C7	0.3 (3)	N3—C19—C20—C21	179.0 (3)
C1—C2—C3—C4	0.2 (5)	C24—C19—C20—C21	55.1 (4)
C2—C3—C4—C5	−0.8 (5)	S1—C19—C24—C23	69.3 (4)
C2—C3—C4—F1	−179.9 (3)	N3—C19—C24—C23	−178.3 (3)
F1—C4—C5—C6	−179.4 (2)	C20—C19—C24—C23	−54.9 (4)
C3—C4—C5—C6	1.5 (4)	C19—C20—C21—C22	−54.2 (5)
C4—C5—C6—C7	180.0 (3)	C20—C21—C22—C23	54.4 (5)
C4—C5—C6—C1	−1.8 (4)	C21—C22—C23—C24	−54.4 (5)
C1—C6—C7—C8	−177.8 (2)	C22—C23—C24—C19	54.7 (5)

### Hydrogen-bond geometry (Å, º)

**Table:**

*D*—H···*A*	*D*—H	H···*A*	*D*···*A*	*D*—H···*A*
N1—H1*A*···O2^i^	0.86 (3)	2.08 (3)	2.903 (4)	160 (2)
N2—H2*A*···O1^ii^	0.85 (2)	2.07 (2)	2.760 (3)	137 (2)
C10—H10*A*···F1^iii^	0.93	2.54	3.453 (5)	167

Symmetry codes: (i) *y*+1, −*x*+*y*+1, *z*+1/6; (ii) *x*−*y*, *x*−1, *z*−1/6; (iii) −*y*, *x*−*y*−1, *z*−1/3.
